# Supplementary material for: Density-dependent sodium-storage mechanisms in hard carbon materials
Source: Chem Sci. 2026 Mar 18;17(19):9570–81. doi: 10.1039/d6sc00030d (PMC13012296; doi:10.1039/d6sc00030d)
Supplement: SC-017-D6SC00030D-s001 [file SC-017-D6SC00030D-s001.pdf]

Supplementary Information (SI) for Chemical Science.
This journal is © The Royal Society of Chemistry 2026

Supplementary information for Density-dependent sodium-storage mechanisms in hard carbon materials

Alexis Front[a,b,*] Tapio Ala-Nissila[b,c], and Miguel A. Caro[a]
[a] Department of Chemistry and Materials Science, Aalto University, Kemistintie 1, 02150 Espoo, Finland
[b] MSP Group, Department of Applied Physics, Aalto University, FI-00076 Aalto, Espoo, Finland and
[c] Interdisciplinary Centre for Mathematical Modelling and Department of Mathematical Sciences, Loughborough University, Loughborough, Leicestershire LE11 3TU, United Kingdom

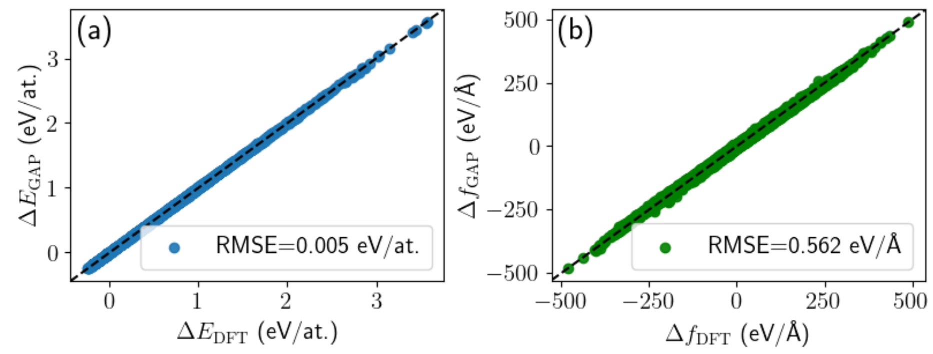

FIG. S1. Accuracy of the Δ-GAP. (a) Scatter plot of GAP versus DFT energies for the training data set after the tenth iteration, (b) same analysis as (a) but for force components.

density (g cm^{-3})	sp (%)	sp^2 (%)	sp^3 (%)
0.7	1.69 (0.60)	97.94 (0.82)	0.33 (0.33)
1.0	1.85 (0.66)	97.75 (0.74)	0.38 (0.25)
1.3	1.33 (0.82)	98.23 (0.94)	0.41 (0.31)
1.6	1.00 (0.37)	98.51 (0.41)	0.47 (0.18)
1.9	1.00 (0.38)	98.49 (0.29)	0.50 (0.31)

TABLE S1. Mean coordination fractions of sp, sp^2, sp^3 for the five HC densities, standard deviations are in parentheses.

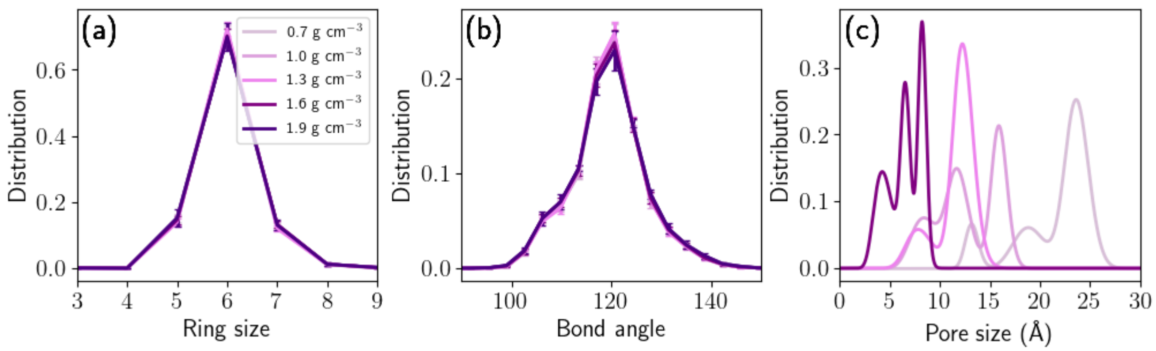

FIG. S2. (a) Mean ring-size distributions, (b) mean bond-angle distributions, and (c) pore-size distributions of each structure used in the sodium insertion simulation. Note that 1.9 g cm^{-3} HC does not present accessible pore. Standard deviation in (a) and (b) are represented with vertical bars.

* Contact author: alexis.front@aalto.fi

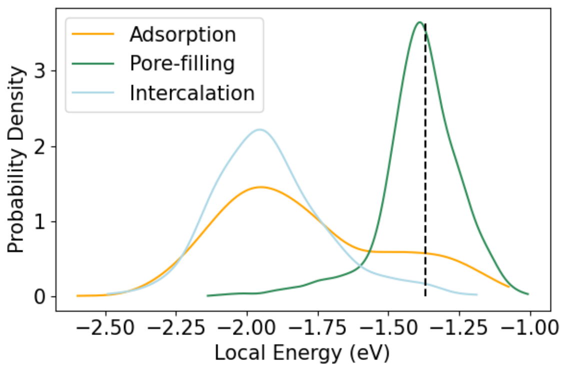

FIG. S3. Local energy distribution of adsorption, pore-filling and intercalation sites. Vertical dotted line represents the sodium metallic bulk energy.

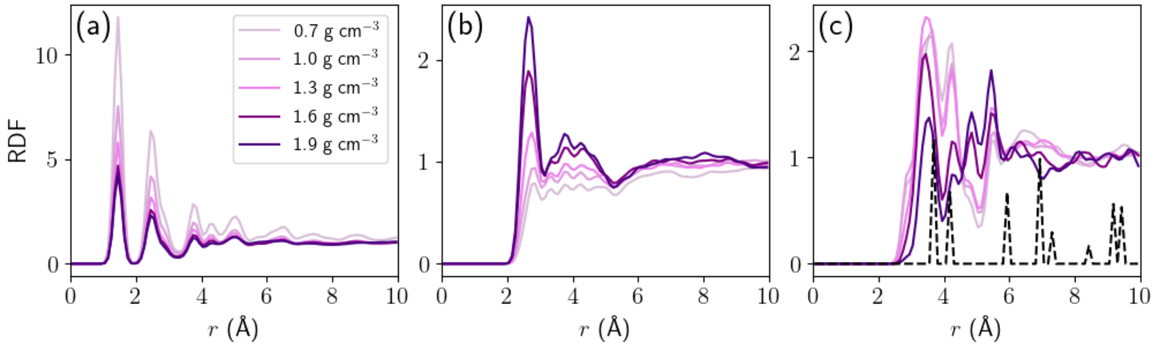

FIG. S4. C-C (a), Na-C (b) and Na-Na (c) partial radial distribution functions (RDF) of the final sodiated structures after sodium insertion simulation. Dotted spectra in (c) corresponds to the bcc sodium bulk.

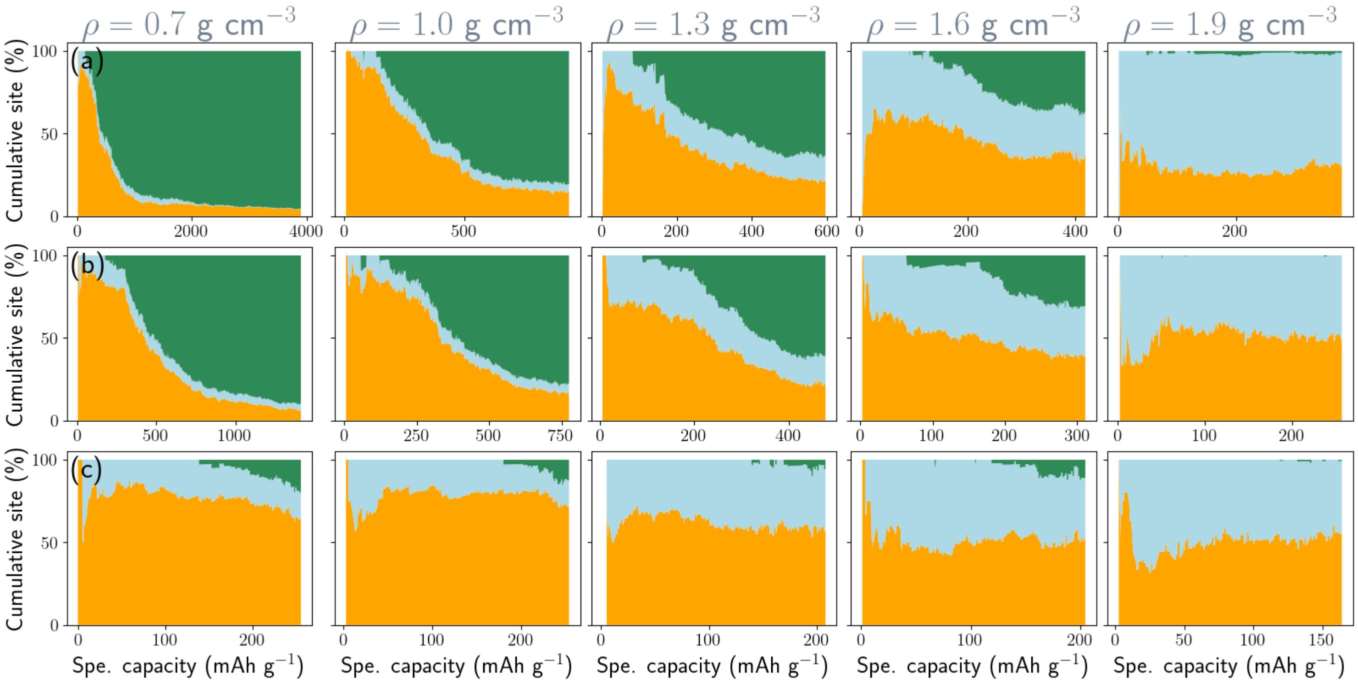

FIG. S5. Cumulative storage capacity of adsorption, intercalation, and pore filling as a function of specific capacity in hard carbon across a density range of 0.7–1.9 g cm^{-3} with a chemical potential of (a) −1.00 eV, (b) −1.24 eV and (c) −1.50 eV. Snapshots of each sites are represented in Fig. S6 and Fig. S7 for (a) and (c) respectively. Color coding: orange = adsorption, blue = intercalation, green = pore-filling.

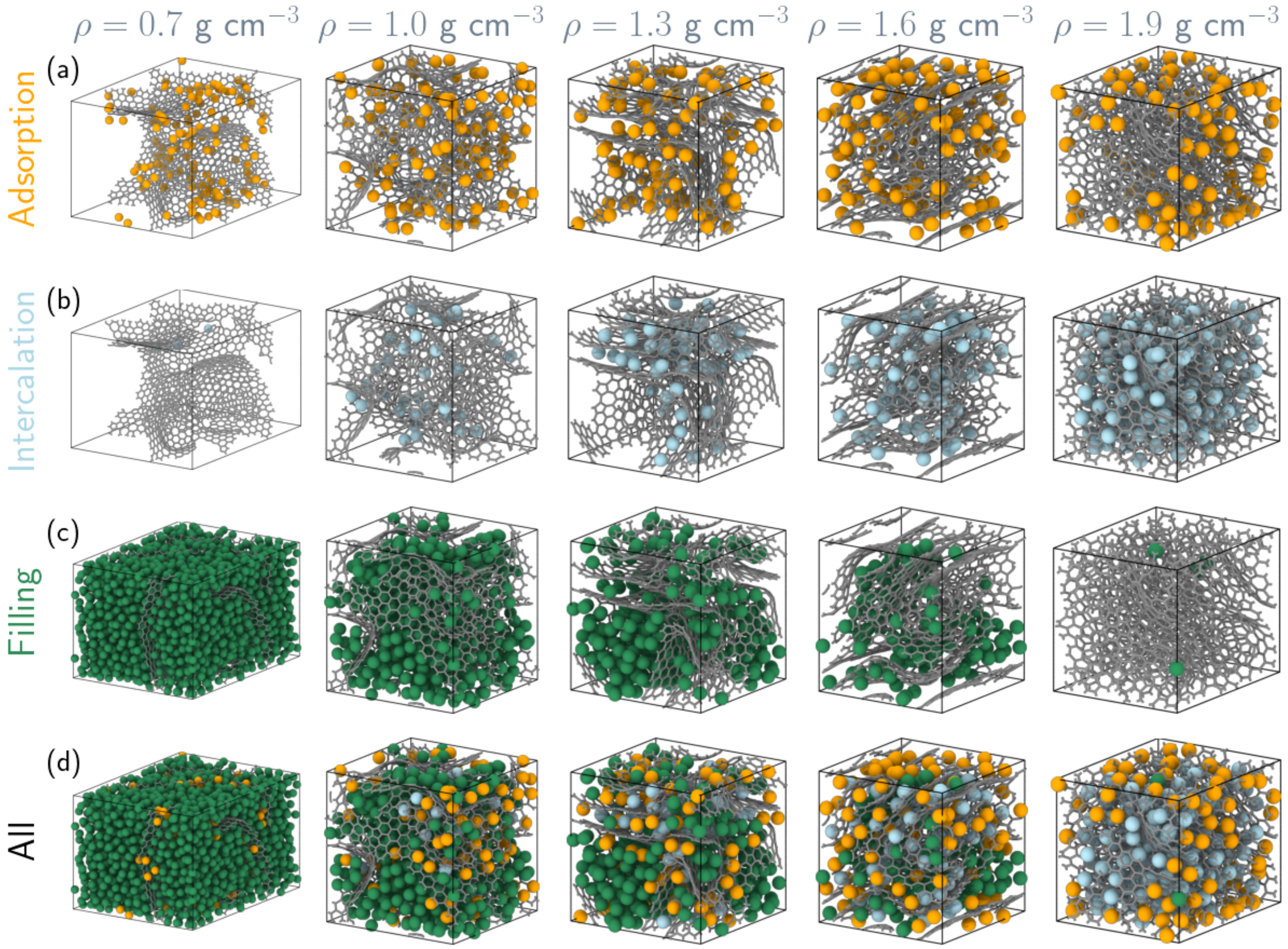

FIG. S6. Snapshots in (a–c) illustrate adsorption, intercalation, and pore-filling sites, respectively. The lower panel (d) shows all three classes of storage sites together. Simulations were performed at 300 K with a chemical potential of $\mu = -1.00$ eV. All snapshots correspond to configurations at the maximum specific capacity. Color coding: orange = adsorption, blue = intercalation, green = pore-filling, and grey = carbon.

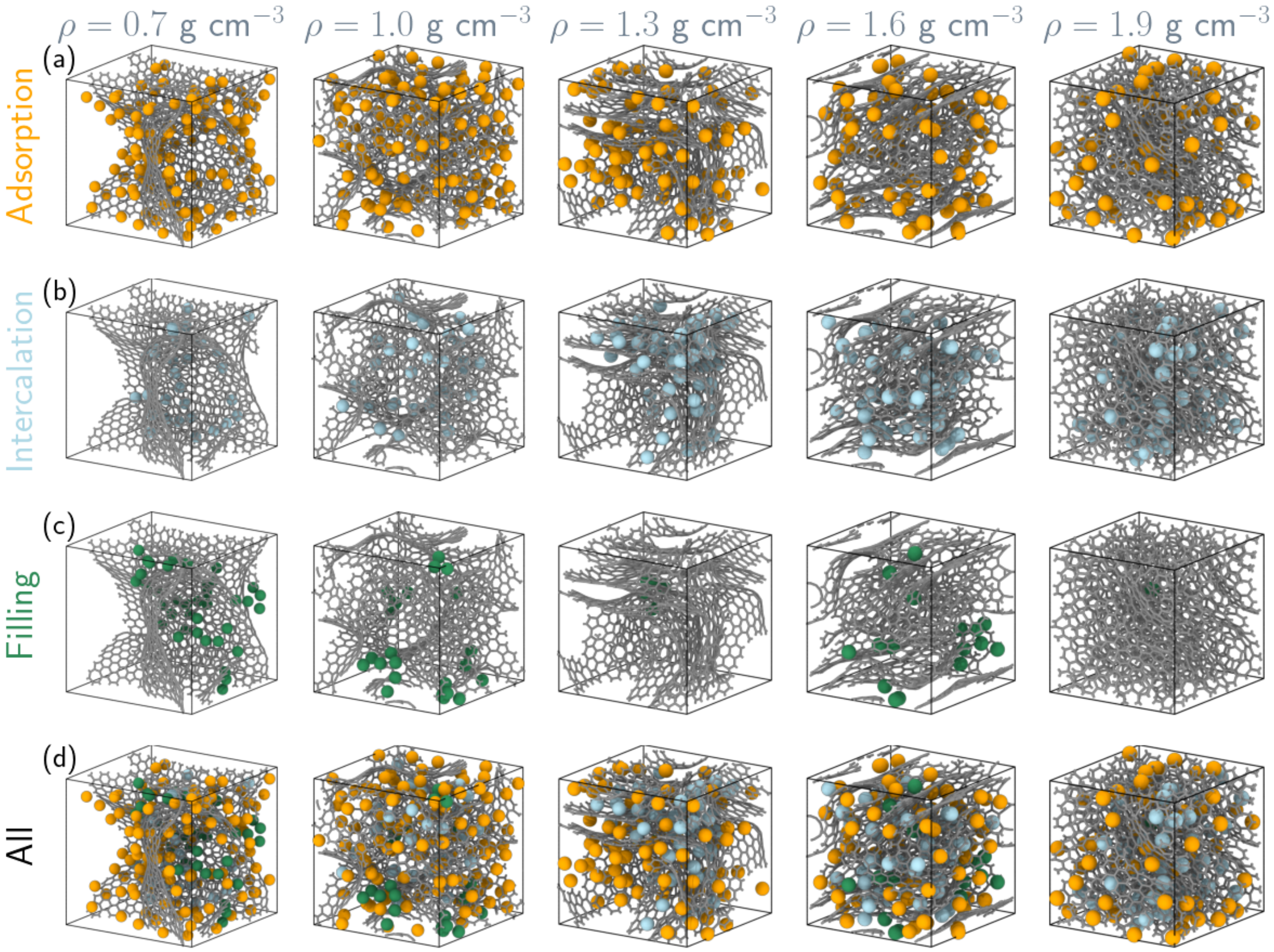

FIG. S7. Snapshots in (a–c) illustrate adsorption, intercalation, and pore-filling sites, respectively. The lower panel (d) shows all three classes of storage sites together. Simulations were performed at 300 K with a chemical potential of $\mu = -1.50$ eV. All snapshots correspond to configurations at the maximum specific capacity. Color coding: orange = adsorption, blue = intercalation, green = pore-filling, and grey = carbon.
